# Pyroptosis is involved in the pathogenesis of human hepatocellular carcinoma

**DOI:** 10.18632/oncotarget.12384

**Published:** 2016-10-01

**Authors:** Qun Chu, Yanan Jiang, Wei Zhang, Chaoqian Xu, Weijie Du, Gulnara Tuguzbaeva, Ying Qin, Anqi Li, Liangshuan Zhang, Guiyuan Sun, Yongqiao Cai, Qiang Feng, Guiyang Li, Yanyao Li, Zhimin Du, Yunlong Bai, Baofeng Yang

**Affiliations:** ^1^ Department of Pharmacology (State-Province Key Laboratories of Biomedicine- Pharmaceutics of China, Key Laboratory of Cardiovascular Research, Ministry of Education), College of Pharmacy, Harbin Medical University, Harbin, Heilongjiang Province, P. R. China; ^2^ Institute of Medical Sciences of Heilongjiang Province, Harbin, P.R. China; ^3^ Central Laboratory of Scientific Research, Bashkir State Medical University, Ufa, Republic Bashkortostan, Russian Federation; ^4^ Institute of Clinical Pharmacology, the Second Affiliated Hospital, Harbin Medical University (Key Laboratory of Drug Development, Universities of Heilongjiang Province), Heilongjiang Province, P. R. China; ^5^ Department of Pharmacology and Therapeutics, Melbourne School of Biomedical Sciences, Faculty of Medicine, Dentistry and Health Sciences, University of Melbourne, Melbourne, Australian

**Keywords:** hepatocellular carcinoma, pyroptosis, caspase-1, berberine

## Abstract

Pyroptosis is a caspase-1 dependent programmed cell death, which is involved in the pathologic process of several kinds of cancers. Loss of caspase-1 gene expression has been observed in prostate and gastric cancers. However, the role of pyroptosis in human hepatocellular carcinoma (HCC) remains largely unknown. The aim of this study was to investigate the involvement of pyroptosis in the pathogenesis of HCC. Our study showed that pyroptosis was inhibited in HCC tissues and cells. Administration of berberine inhibited the viability, migration and invasion capacity of HepG2 cells through the induction of pyroptosis both in vitro and in vivo, which was attenuated by caspase-1 inhibitor Ac-YVAD-CMK. Conclusively, pyroptosis is involved in the pathogenesis of HCC, and may be a new neoplastic target for the treatment of HCC.

## INTRODUCTION

Liver cancer is the second-commonest cause of cancer related death, and accounted for 746,000 world deaths in 2012 [1]. Human hepatocellular carcinoma (HCC) arises from hepatocytes and represents the most frequent type of primary liver cancers. Although, a large amount of genomic and epigenetic alterations has been identified, mainly focused on apoptosis, autophagy and hepatitis virus, the treatment of HCC is still a conundrum [2–4]. Therefore, it is very important to clarify the molecular mechanisms of HCC and find novel therapeutic targets for this malignancy.

Pyroptosis is a form of caspase-1 dependent programmed cell death described in myeloid cells. Activation of caspase-1 distinguishes pyroptosis from other kinds of cell death. In this process, cells recognize certain harmful signals, produce cytokines, swell, burst, and ultimately die [5]. Pyroptosis has been demonstrated to aggravate diabetic cardiomyopathy, hepatic fibrosis and diabetes [6–8]. For cancer cells, the activation of pyroptosis may promote cell death and thus exert anticancer properties. The loss of caspase-1 gene expression was found in human prostate cancer [9, 10]. Moreover, caspase-1 deficiency enhances inﬂammation- induced colorectal tumor formation in mice [11]. However, little is known regarding the role of pyroptosis in HCC.

Berberine is a natural isoquinoline alkaloid, which is well known for its excellent antimicrobial activity and mild side effects [12]. Recently, an increasing number of studies has proved that berberine is a promising agent for cancer treatment [13]. It has been reported that berberine can induce both autophagy and apoptosis in HCC cells [14, 15]. In our preliminary experiments, we observed that berberine could activate caspase-1. We therefore set out the present study and explored the role of pyroptosis in the progression of HCC and the involvement of pyroptosis as a possible mechanism by which berberine induces HCC.

## RESULTS

### Loss of caspase-1 expression in HCC

**Figure 1:**
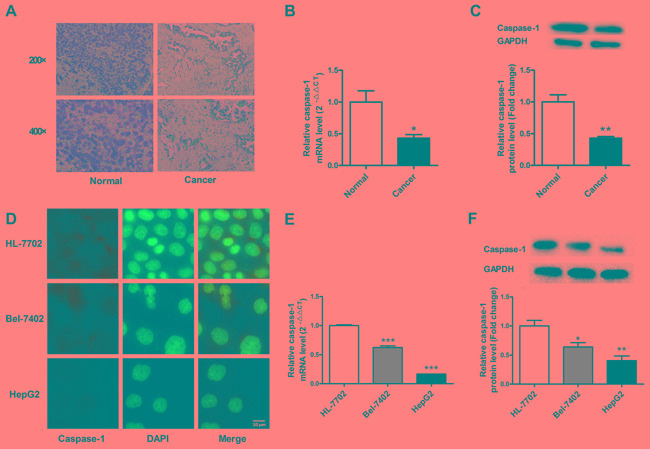
Loss of caspase-1 expression in human hepatocellular carcinoma (HCC) tissues and cells **A.** The immunohistochemical staining of caspase-1 in HCC and adjacent normal tissues. **B** & **C.** The expression of caspase-1 mRNA and protein in HCC and adjacent normal tissues. **D.** The immunofluorescence staining of caspase-1 in HL-7702, Bel-7402 and HepG2 cells. **E** & **F.** The expression of caspase-1 mRNA and protein in HL-7702, Bel-7402 and HepG2 cells. GAPDH served as an internal control. For panel B and C, *P < 0.05 versus Normal, **P < 0.01 versus Normal. For panel E & F, *P < 0.05 versus HL-7702, **P < 0.01 versus HL-7702, ***P < 0.001 versus HL-7702; *n* = 3.

The expression of caspase-1 in HCC was determined in human tissues and cell lines. A significant decrease in caspase-1 immunostaining was observed in HCC tissues from patients (Figure 1A). To further evaluate the expression of caspase-1, real-time PCR and western blot were performed. The results showed that caspase-1 mRNA and protein levels were downregulated in HCC tissues relative to adjacent normal tissues (Figure 1B & 1C). A similar phenomenon was observed in HCC and hepatocyte cell lines: the expression of caspase-1 in Bel-7402 and HepG2 cells was lower than that in HL-7702 (Figure 1D,E & 1F). These findings suggested that loss of caspase-1 expression is involved in the pathogenesis of HCC.

### Berberine induces pyroptosis in HepG2 cells

**Figure 2:**
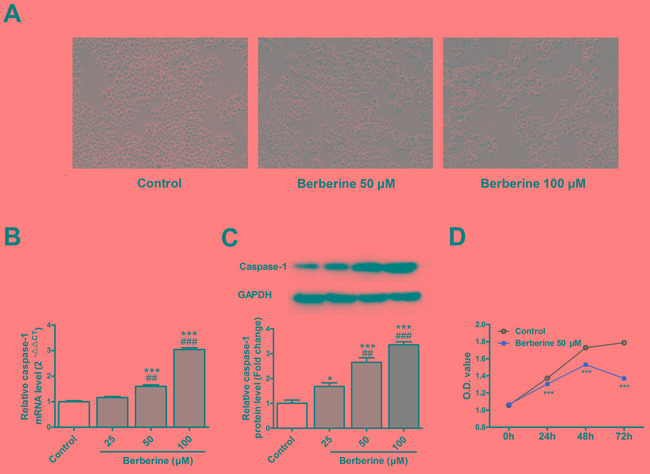
Berberine induces pyroptosis in HepG2 cells **A.** Decrease in cell number and swelling of cells in HepG 2 cells treated with berberine. **B.** Expression alterations of caspase-1 mRNA in HepG2 cells treated with berberine. **C.** Changes of caspase-1 protein levels in HepG2 cells treated with berberine. **D.** The effect of berberine on the cell viability of HepG2 cells. GAPDH served as an internal control. *P < 0.05 versus Control, ***P < 0.001 versus Control; ^##^*P* < 0.01 versus Berberine 25 μM, ^###^*P* < 0.001 versus Berberine 25 μM; For panel A, B & C *n* = 3; For panel D, *n* = 6.

The cell number was decreased and cell swelling was observed after treatment with berberine (Figure 2A). Berberine increased the mRNA and protein expression of caspase-1 in HepG2 cells in a concentration-dependent manner with statistical significance starting from 50 μM (Figure 2B & 2C). Thus, this concentration was used for the subsequent experiments. We also found that berberine decreased the viability of HepG2 cells (Figure 2D).

### Caspase-1 inhibitor attenuates the effects of berberine in HepG2 cells

**Figure 3:**
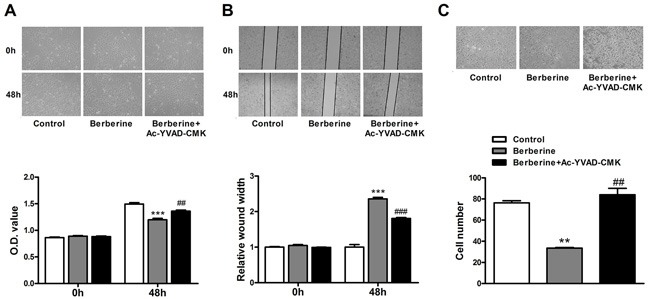
Caspase-1 inhibitor attenuates the inhibitory effects of berberine on proliferation, migration and invasion of HepG2 cells **A.** Alterations of viability of HepG2 cells, as revealed by MTS assay. **B.** Migration distance of HepG2 cells. **C.** Altered number of invasive HepG2 cells. **P < 0.01 versus Control, ***P < 0.001 versus Control; ^##^*P* < 0.01 versus Berberine, ^###^*P* < 0.001 versus Berberine; *n* = 2-4.

**Figure 4:**
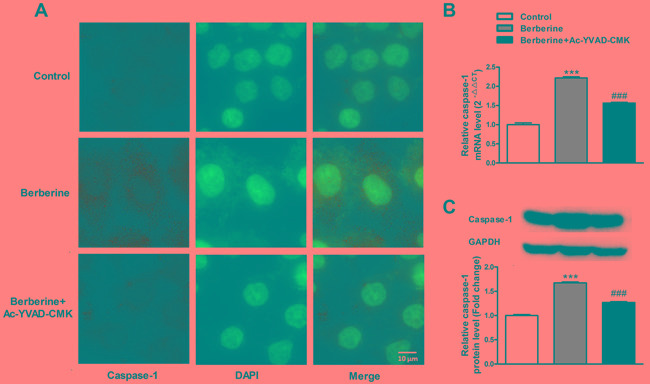
Caspase-1 inhibitor Ac-YVAD-CMK reverses the inhibitory effect of berberine on caspase-1 expression in HepG2 cells **A.** The immunofluorescence staining of caspase-1 in HepG2 cells. **B.** The expression of caspase-1 mRNA in HepG2 cells. **C.** The expression of caspase-1 protein in HepG2 cells. GAPDH served as an internal control. ***P< 0.001 versus Control; ^###^*P*< 0.001 versus Berberine; *n* = 3.

MTS assay, scratch-wound assay and Boyden Chamber cell invasion assay were conducted to determine the effects of berberine on HepG2 cell. The results showed that berberine inhibited the viability, and migration and invasion capacity of HepG2 cells, and these effects were attenuated by caspase-1 inhibitor Ac-YVAD-CMK (Figure 3). The expression upregulation of caspase-1 induced by berberine treatment was also effectively reversed by caspase-1 inhibitor (Figure 4). These results suggest the effects of berberine were at least partially through activation of caspase-1.

### Caspase-1 inhibitor attenuates the effects of berberine in xenograft mouse model

**Figure 5:**
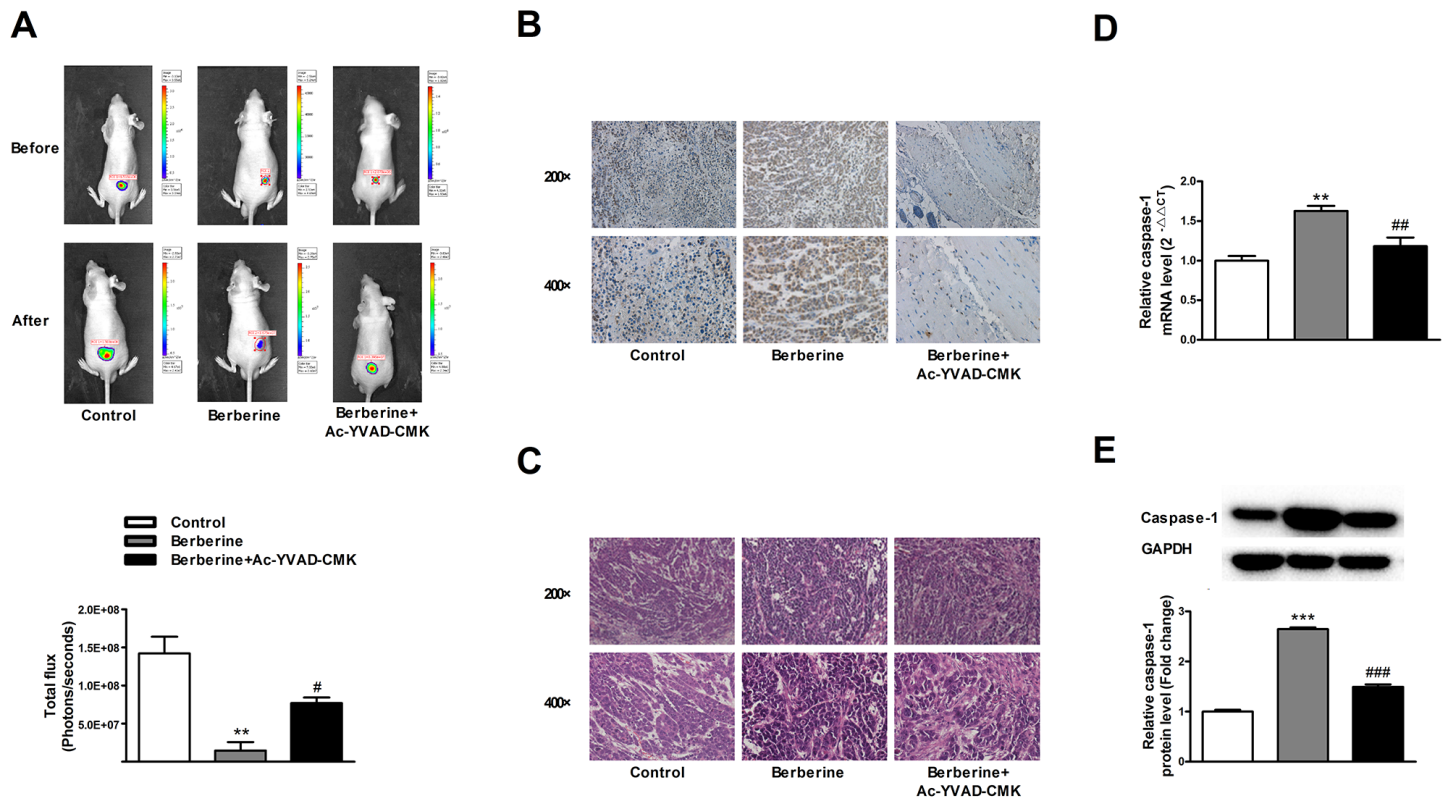
Caspase-1 inhibitor abolishes the beneficial effects of berberine on xenograft tumors in nude mice **A.** Tumor volume of mice. **B.** The histopathological changes in tumors. **C.** The immunohistochemical staining of caspase-1 in tumors. **D.** The expression of caspase-1 mRNA in HepG2 cells. **E.** The expression of caspase-1 protein in HepG2 cells. GAPDH served as an internal control. **P < 0.01 versus Control, ***P < 0.001 versus Control; ^##^*P*< 0.01 versus Berberine, ^###^*P* < 0.001 versus Berberine; *n* = 3.

We then carried out the *in vivo* experiments using a xenograft mouse model to verify the *in vitro* observations. The tumor volume was significantly decreased after treatment with berberine, compared with the control group, and caspase-1 inhibitor attenuated the effects of berberine (Figure 5A). After the measurements, the mice were sacrificed and the tumor tissues were dissected for histology, immunohistochemical, real-time PCR and western blot analyses. As shown in Figure 5B, berberine triggered an inflammatory reaction of HCC tissues, which was attenuated by caspase-1 inhibitor. Meanwhile, berberine significantly increased the expression of caspase-1, which was reversed by caspase-1 inhibitor (Figure 5C,D & 5E).

## DISCUSSION

HCC is considered to be one of the most common malignancies worldwide. However, current medication is not sufficient to resolve the major concern about the long-term treatment efficacy. Therefore, uncovering the molecular pathogenesis of HCC and finding new therapeutic targets are vitally important.

Pyroptosis is a newly discovered way of programmed cell death, and it is associated with activation of caspase-1 to induce cancer cell death. Whilst activation of pyroptosis has been shown to elicit beneficial effects against many types of cancers, its potential role in HCC largely remained a riddle. The present study provided the evidence for the involvement of pyroptosis in HCC, as indicated by loss of caspase-1 expression in human HCC and adjacent normal tissues, as well as in HCC and hepatocyte cell lines. Moreover, berberine restored the abnormal downregulation of caspase 1, mitigating the viability, migration and invasion capacity of HepG2 cells, and these effects were attenuated by caspase-1 inhibitor Ac-YVAD-CMK. These findings indicate that pyroptosis process is inactivated in HCC and berberine reactivated the program.

It has been widely accepted that berberine possesses inhibitive effects on breast, lung, prostate and liver cancers [15–18]. However, the molecular mechanisms underlying the anti-cancer efficacy of berberine have not been fully elucidated. Several mechanisms have been delineated to explain the anticancer properties of berberine, of which activation of apoptosis is the best characterized event. Previously, caspase-1 was implicated in the execution of apoptosis. We found here that berberine exerts inhibitory effects on HCC both in vitro and in vivo, consistent with the published results [19–21]. Our data strongly suggest that the anti-HCC efficacy of berberine could be ascribed at least partially to its ability to stimulating pyroptosis via rescuing the expression of caspase-1. Pyroptosis could be either beneficial or detrimental depending on different pathological conditions. As for cancer, activation of caspase-1 can increase the secretion of pro-inflammatory cytokines to exert carcinogenesis effects or induce cell death to exert carcinostasis effect. In the present study, we found that the induction of pyroptosis exerted inhibitive effect on HCC.

In summary, three important findings are presented in the current study. First, inhibition of pyroptosis was involved in the pathogenesis of HCC. Second, activation of caspase-1 offered a therapeutic potential against HCC. Last, berberine produced anti-HCC effects at least partially through the activation of caspase-1.

## MATERIALS AND METHODS

### Patient samples

Three pairs of HCC and adjacent normal tissues were obtained from the Third Affiliated Hospital of Harbin Medical University. The experiments were carried out in accordance with The Code of Ethics of the World Medical Association (Declaration of Helsinki) for experiments including human. This study was approved by the Ethics Committee of Harbin Medical University, China. Written informed consent was obtained from each participant after a full explanation. The tissues were collected at the time of surgical resection.

### Cell culture

HepG2 and HL-7702 cell lines were kind gifts by Professor Gao Xu (Department of Biochemistry and Molecular Biology, Harbin Medical University). Bel-7402 cell line was purchased from iCell bioscience (Shanghai, China). HepG2, HL-7702 and Bel-7402 cells were maintained in RPMI-1640 containing 10% FBS and 1% penicillin/streptomycin. The cells were incubated at  37°C in a humidified chamber containing 5% CO_2_.

### MTS assay

To detect the effect of berberine on cell viability, HepG2 cells were seeded into a 96-well culture plate, and treated with berberine chloride (Sigma, MO, USA) (50μM) or in combination with caspase-1 inhibitor Ac-YVAD-CMK (100 μM). After treatment, cell viability was assessed using the CellTiter 96® AQueous One Solution Reagent (Promega, Madison, WI, USA) according to the manufacturer's instructions.

### Scratch-wound assay

Cells were seeded into a 12-well culture plate and the cell monolayer was scratched in a straight line with a sterile 10-μL pipette tip. In order to remove the debris and smooth the edge of the scratch, culture medium was removed and wells were washed three times with the medium. Berberine and caspase-1 inhibitor Ac-YVAD-CMK were then added to culture medium. The width of the open area was measured 48 h later. The distance of wound closure was used to estimate the migration ability.

### Boyden chamber cell invasion assay

After pretreatment with berberine for 24 h, cells were harvested and seeded to an invasion chamber (BD BioCoat, CA, USA) at 1.5×10^4^ cells per well in serum-free medium, and then incubated at 37°C for another 24 h. The invaded cells were fixed with methanol and stained with Giemsa. Cell numbers were counted under a light microscope.

### Xenograft mouse model

The experimental protocols were approved by the Ethic Committee of Harbin Medical University, China. Use of animals was conformed to the National Institutes of Health guide for the care and use of laboratory animals published by the US National Institutes of Health (NIH Publication No. 85–23, revised 1996).

BALB/c-nude male mice of 5 to 6 weeks old weighing 18-20 g were used. The mice were housed with a regular 12-h light/12-h dark cycle with *ad libitum* access to standard rodent chow diet and were kept in a pathogen-free environment. For in vivo tracking, HepG2 cells were stably transfected with firefly luciferase. The HepG2 cells (1×10^7^cells suspended in 100μL serum-free DMEM) were injected subcutaneously into the back of mice. Eight days post-implantation, the mice were randomly divided into three groups (*n* = 6 for each group) and fed by oral gavage with saline, berberine (20 mg·kg^-1^·d^-1^), or berberine and intraperitoneal caspase-1 inhibitor Ac-YVAD-CMK (0.1 mg·kg^-1^·d^-1^). Tumor volume was monitored by luciferase activity in HepG2 cells, and the emitted photons from the target site penetrated through the mammalian tissue and could be externally detected and quantified using a sensitive light imaging system. Mice were euthanized 21 days after treatment and the tumors were isolated for further detection.

### Hematoxylin and eosin (HE) staining

Tumor tissues were fixed in 4% paraformaldehyde followed by dehydration. The processed samples were embedded in paraffin and cut into 5-μm thick sections using tissue-processing equipment. The sections were deparaffinized and stained with HE for histological analysis.

### Immunohistochemistry

HCC and adjacent tissues were fixed with 4% buffered paraformaldehyde, dehydrated and embedded in paraffin. Five-μm sections were deparaffinized, rehydrated, and rinsed in distilled water. Antigen unmasking was carried out by microwave heating in citrate buffer for 20 minutes. All sections were immunostained with the primary antibody against caspase-1 (cell signaling, MA, USA) at 4°C overnight. After incubation with the secondary antibody, the sections were stained with diaminobenzidine.

### Immunofluorescence staining

Cultured cells were fixed with 4% paraformaldehyde at room temperature for 20 min. Nonspecific binding was blocked for 2 h by goat serum and cells were subsequently incubated with primary antibody (1:50) at 4°C overnight. Then the cells were washed and incubated with second antibody for 1 h. The images were captured using a fluorescence microscope.

### Real-time PCR

The total RNA samples were extracted from cells or tumor tissues using the Trizol reagent (Roche, IN, USA). Total RNA for 500 ng was reverse transcribed to cDNA using Reverse Transcription Master Kit (Toyobo, Osaka, Japan) according to the manufacturer's instructions. Real-time PCR was performed on ABI 7500 fast system (Applied Biosystems, CA, USA) using SYBR Green I (Toyobo, Osaka, Japan). The sequences of the primer pairs are as follows. Caspase-1: Forward 5’-ACACGTCTTGCCCTCATTATCT-3’, Reverse 5’-ATAACCTTGGGCTTGTCTTTCA-3’; GAPDH : Forward 5’-ATCACTGCCACCCAGAAGAC-3’, Reverse 5’-TTTCTAGACGGCAGGTCAGG-3’. GAPDH served as an internal control. The relative quantification of gene expression was determined using the 2^-ΔΔCT^ method.

### Western blot analysis

Total protein was extract from cells or tissues. The suspension was subjected to 10% acrylamide gel electrophoresis (SDS-PAGE) followed by electrotransfer onto a nitrocellulose filter. After blocked with 5% (w/v) non-fat milk dissolved in PBS for 2 h, the membranes were incubated at 4°C overnight with primary antibodies of caspase-1 (Cell Signaling, MA, USA) and GAPDH (ZSGB-BIO, Beijing, China), followed by incubation with HRB labeled goat anti-mouse IgG or anti-rabbit IgG (1:1000) (ZSGB-BIO, Beijing, China) for 1 h. Western blotting bands were quantified using Quantity One software.

### Statistical analysis

Data are expressed as mean ± standard error of mean (mean ± SEM) and were analyzed with SPSS 13.0 software. Statistical comparisons between two groups were performed using Student's *t*-test. Statistical comparisons among multiple groups were performed using analysis of variance (ANOVA). A two-tailed *P* < 0.05 was considered statistically significant. Graphs were generated using Graphpad Prism 5.0.

## SUPPLEMENTARY MATERIALS FIGURE


