# Supplementary material for: Pyroptosis is involved in the pathogenesis of human hepatocellular carcinoma
Source: Oncotarget. 2016 Oct 1;7(51):84658–65. doi: 10.18632/oncotarget.12384 (PMC5356689; doi:10.18632/oncotarget.12384)
Supplement: Supplementary file 1 [file oncotarget-07-84658-s001.pdf]

Pyroptosis is involved in the pathogenesis of human hepatocellular carcinoma

SUPPLEMENTARY FIGURE

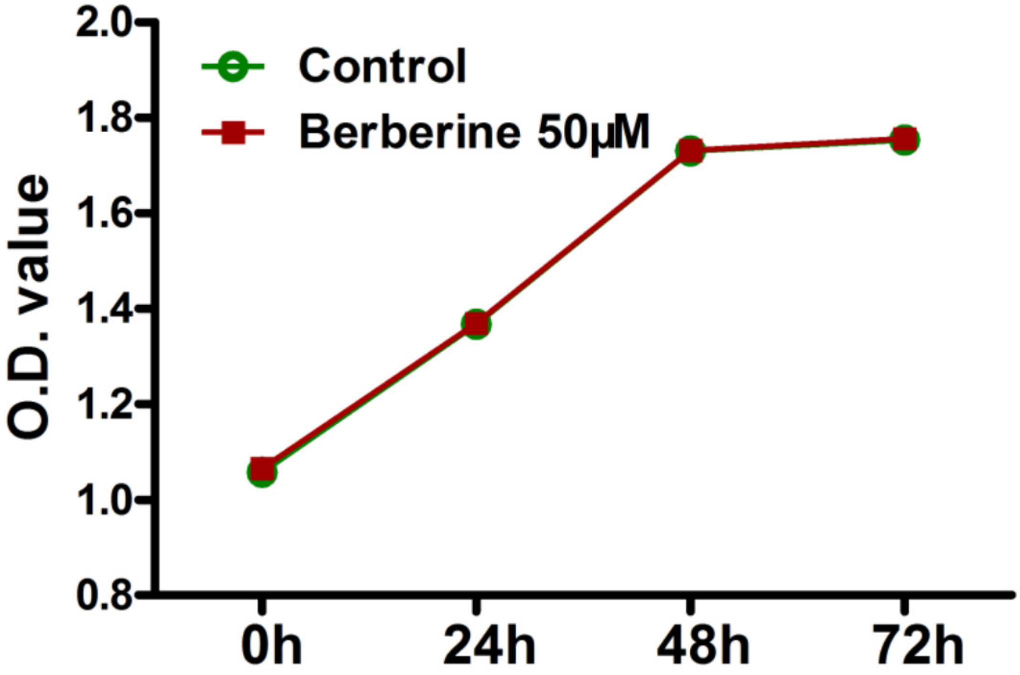

Supplementary Figure S1: The growth curve of HL-7702 cell treated with berberine. *n* = 6.
